# Cascade of care and factors associated with virological suppression among HIV-positive persons linked to care in the Test and Keep in Care (TAK) project

**DOI:** 10.1007/s15010-018-1154-0

**Published:** 2018-05-21

**Authors:** Justyna D. Kowalska, Magdalena Ankiersztejn-Bartczak, Leah Shepherd, Amanda Mocroft

**Affiliations:** 1 Department of Adults’ Infectious Diseases, Medical University of Warsaw, Ul. Wolska 37, 01-201 Warsaw, Poland; 2 Hospital for Infectious Diseases, HIV Out-Patient Clinic, Warsaw, Poland; 3 Foundation of Social Education (FES), Warsaw, Poland; 4 University College Medical School, London, UK

**Keywords:** HIV, Linkage, Viral suppression, Cascade of care, Antiretroviral therapy

## Abstract

**Introduction:**

Early treatment remains the most effective HIV prevention strategy; poor linkage to care after HIV diagnosis may compromise this benefit. We sought to better understand patient characteristics and their association with virological suppression (VS) following cART initiation.

**Methods:**

The TAK project collects pre-linkage to care and clinical data on patients diagnosed with HIV in voluntary testing facilities in central Poland. Data collected for persons diagnosed in 2010–2013 were linked with HIV clinic records. Individuals linked to care who commenced cART were followed from until the earliest of first VS (HIV RNA < 50 copies/ml), last visit, death or 6 January 2016. Cox-proportional hazard models were used to identify factors associated with first viral suppression.

**Results:**

232 persons were HIV positive, 144 (62%, 95% CI 55, 68%) linked to care, 116 (81% of those linked to care, 95% CI 73, 87%) started cART during follow up, of which 113 (97%, 95% CI 93, 99%) achieved VS. Non-PI based regimen (for integrase inhibitors aHR: 5.03: 1.90, 13.32) and HLA B5701-positive (aHR: 3.97: 1.33, 11.85) were associated with higher chance of VS. Unknown syphilis status (aHR: 0.27: 0.13, 0.57) and higher HIV RNA (aHR a tenfold increase: 0.56: 0.42, 0.75) remained associated with lower chance of VS.

**Conclusions:**

Although a low proportion of persons were linked to care, almost all those linked to care started cART and achieved rapid VS. The high rates of VS were irrespective of prior HIV-associated risk behaviours. Linkage to care remains the highest priority in prevention strategies in central Poland.

## Introduction

Combination antiretroviral therapy (cART) remains the most effective HIV prevention strategy, if persons with HIV infection are identified, linked to care and receive effective treatment [1, 2]. To significantly reduce the impact of the HIV epidemic, UNAIDS has set the target of achieving at least 90% of persons with HIV diagnosed, 90% of those diagnosed to be linked to care and 90% of those linked to care on effective cART by 2020 [3, 4]. The cascade of care is a straightforward and simple summary of the sequential stages of HIV medical care for people living with HIV; and can be used to identify stages in care to be targeted to curb the epidemic across the whole range of interventions [5, 6]. While the left side of the cascade describes the actions taken for screening for HIV, the right side provides information on effectiveness of treating those who are diagnosed. Taken together, the cascade of care has the power to show the effectiveness of collaboration between public health institutions and medical care providers. This area has been identified as a weak spot for many European countries, especially for Central and Eastern Europe [7].

In our earlier work, we have identified that over 40% of the patients diagnosed in voluntary counselling and testing facilities (VCTs) in central Poland were not linked to care, and this situation did not change during the 4 years of observation [8, 9]. In the present study, our aim was to construct a cascade of care for central Poland. In addition, we investigate the rate of viral suppression, as well as pre-linkage and clinical factors related to it.

## Materials and methods

The TAK project collects information on patients diagnosed with HIV in VCTs in central Poland, follows their linkage to care and ongoing routine clinical care [10]. Data collected for persons diagnosed in 2010–2013 in VCTs were linked with HIV clinic records using the western blot test number as a unique identifier. This allows for persons who test positive for HIV to be followed throughout linkage to care and ongoing routine clinical care following their diagnosis. Persons linked to care are managed in accordance with the Polish AIDS Society recommendations [11].

### Cascade of care

To set the scene, a complete cascade of care was constructed using the TAK information. The proportion of people living with HIV in Poland (both diagnosed and undiagnosed) is unknown; therefore, the first stage in the cascade was considered to be those who tested positive for HIV at the VCTs (100%). The subsequent stages of the cascade are shown as both percentages according to those who tested positive for HIV (fixed denominator), as well as step wise percentages, i.e., the percentages of HIV-positive people who were linked to care, of those linked to care who started cART, and of those who started cART who achieved VS.

### Factors associated with the final stage of the cascade: virological suppression in those who started cART

For the second component of the project, baseline was defined as the date of first cART, defined as three or more antiretroviral drugs including integrase inhibitor (InSTI), protease inhibitor (PI) or non-nucleoside reverse transcriptase inhibitors (NNRTI). People were followed from baseline until first viral suppression, defined as HIV RNA < 50 copies/ml, or censored at last visit, death or 6 Jan 2016, the administrative censoring date. Persons who did not have a HIV RNA and CD4+ count measured at cART start were excluded.

The distribution of variables at baseline were shown according to those who did and did not achieve viral suppression. Frequencies and percentages with Chi-squared tests are shown for categorical variables, and median and interquartile range (IQR) with Wilcoxon–Mann–Whitney tests are shown for continuous variables. Kaplan–Meyer curves were used to investigate the time to viral suppression in those who started cART overall as well as stratified by year of starting cART.

Univariate and multivariate Cox-proportional hazard models were used to identify factors measured at time of cART initiation which were associated with higher chance of viral suppression. Factors considered for inclusion included age, gender, nationality (Polish vs other), education (high vs lower/unknown), sexual orientation, HIV test location (VCTs vs other), number of HIV tests within the last year, partner’s HIV status, partner’s testing status, number of partners in last year and in the lifetime, condom use with stable partner, condom use with casual partners, having sex under the influence of drugs or alcohol, time to linkage to care, lymphocyte CD3+, CD4+ and CD8+ count, CD4+/CD8+ ratio, year of first cART, HIV RNA at starting cART, syphilis, anti-HCV antibodies and anti-HBc total antibodies (HCV and HBV) status, HIV subtype, HLA B5701 genotype (positive vs negative) and cART regimen. Factors with *P* < 0.1 in univariate models were included in multivariate models, which were additionally adjusted for age. Factors with a frequency of < 5 in any level were not considered for inclusion in univariate or multivariate models.

Basic characteristics of those stopped treatment during follow up were compared according to drug class. Those who stopped were split according to those who switched regimens (defined as stopping the first regimen and starting the second within 7 days) and those who did not restart after stopping. The number and percent of people who stopped their initial regimen as well as the reason for stopping were reported.

The study was approved by the Bioethical Committee of the Medical University of Warsaw (AKBE/99/16). Informed consents were not collected due to the anonymous character of VCT testing.

## Results

**Fig. 1:**
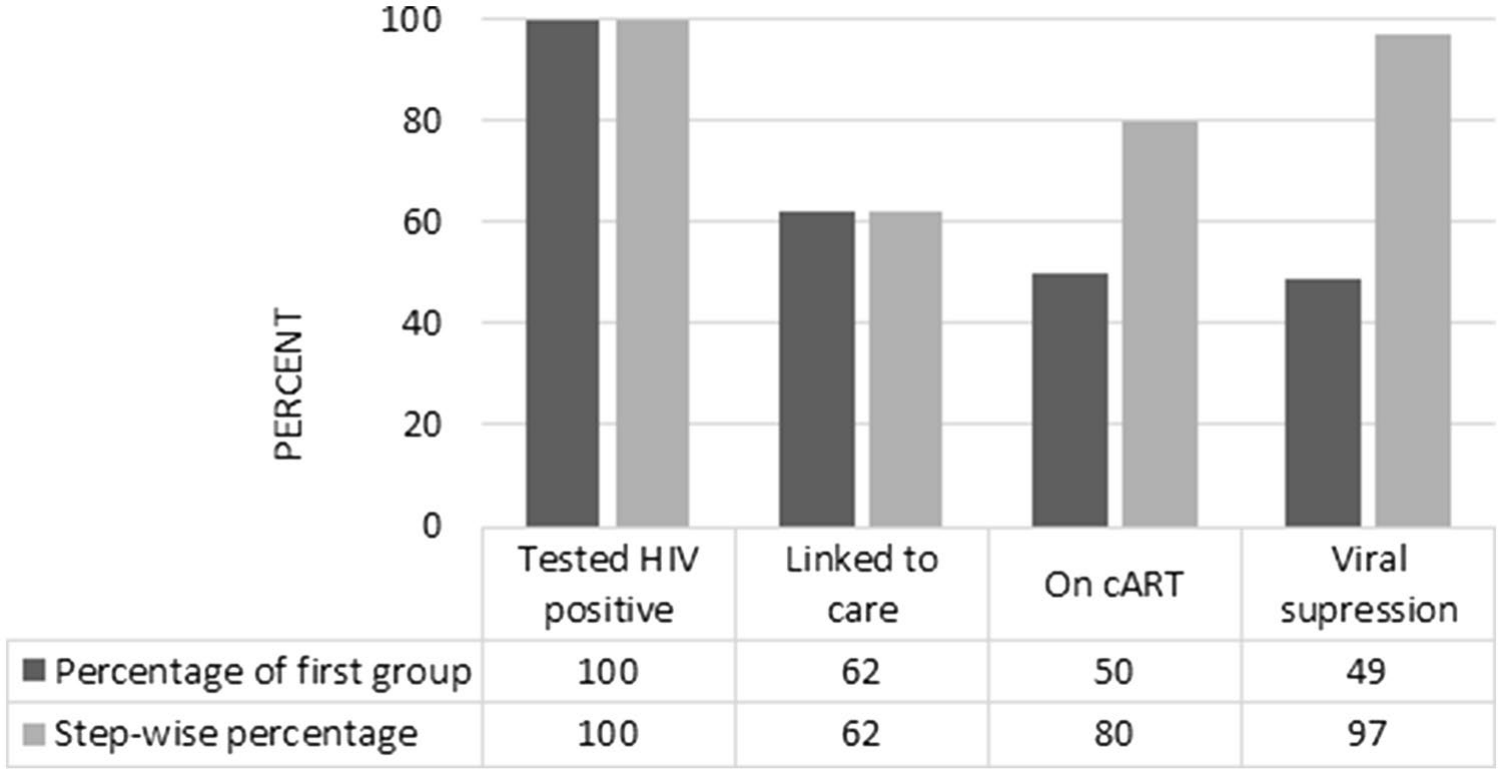
Cascade of care for TAK project

**Fig. 2:**
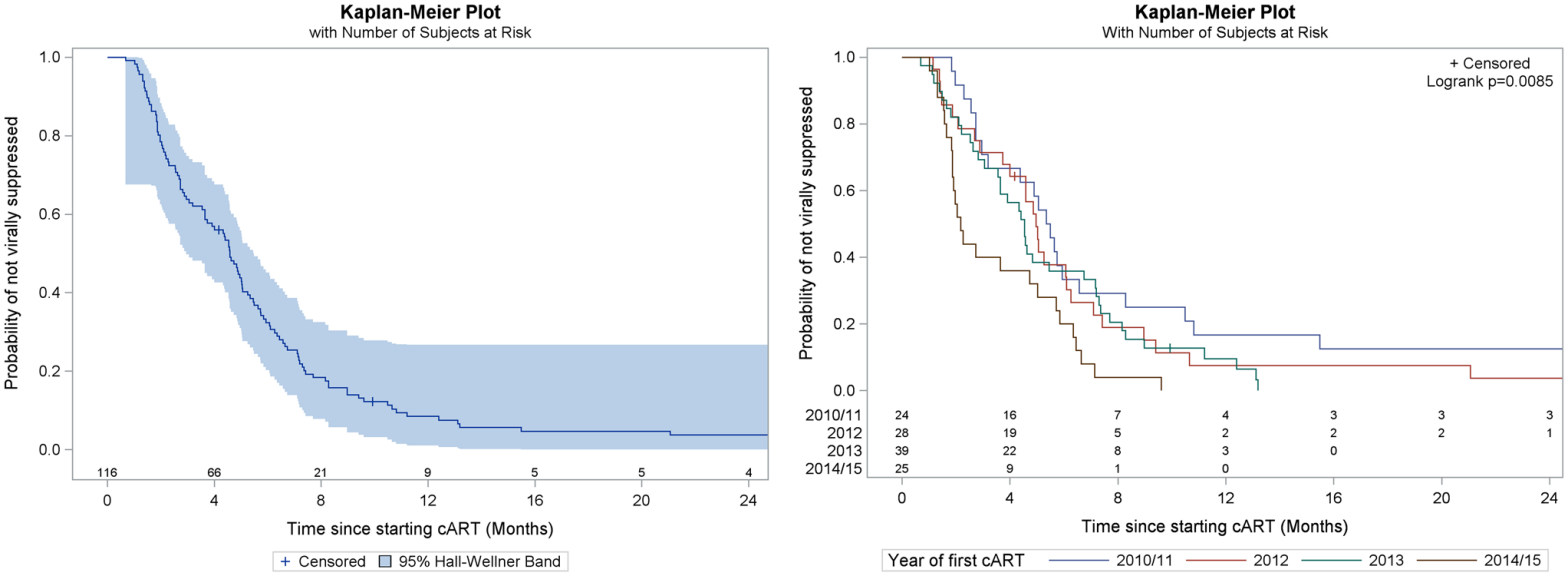
Kaplan–Meier survival curve for time to viral suppression in those who start cART presented for all (left side) and by year of starting cART (right side)

Two hundred and thirty-two persons were tested HIV positive at VCTs and 144 (62%, 95% CI 55, 68) linked to care (Fig. 1). Of those linked to care, 116 (81% 95% CI 73, 87) started cART during follow up, with a median time from linkage to care to starting cART of 6 (IQR 3–9) months. One hundred and thirteen (97%, 95% CI 93, 99%) persons achieved viral suppression in a median time of 5 (IQR 4–5) months (Fig. 2).The median survival time to viral suppression has significantly reduced in more recent calendar years, from a median of 5.42 (95% CI 2.95, 4.89) months in 2010/11, through 4.96 (95% CI 3.74, 6.74) in 2012 and 4.54 (95% CI 3.06, 6.74) in 2013 to 2.17 (95% CI 1.87, 5.03) months in 2014/15 (Fig. 2, *P* = 0.01).

Of those who started cART 111 (96%) were men, 98 (85%) were of homosexual orientation, 26 (22%) had a positive venereal disease research laboratory test (VDRL) at baseline, 34 (29%) had a partner who had never tested for HIV, 33 (28%) had a HIV-positive partner, 66 (57%) reported always using condoms with casual partners, and 24 (21%) reported having sex on drugs or alcohol. The median age was 32 (IQR 28–37) years, lymphocyte CD4+ count was 353 (260–416) cells/µl and HIV RNA was 4.5 (3.9–5.1) log10 copies/ml at first starting cART (Table 1).

**Table 1:** Characteristics of persons who were tested HIV positive at VCT, linked to care and started cART stratified by viral suppresion

Factors	Total *N* = 116	Viral suppression No *N* = 3	Viral suppression Yes *N* = 113	*P* value
	Number (%)			
Male gender	111 (95.7)	3 (100)	108 (95.6)	0.981
Polish nationality	112 (96.6)	3 (100)	109 (96.5)	0.983
High education	94 (81.0)	3 (100)	91 (80.5)	0.959
Sexual orientation				
Heterosexual	12 (10.3)	0 (0.0)	12 (10.6)	0.998
Homosexual	98 (84.5)	3 (100)	95 (84.1)	
Bisexual	6 (5.2)	0 (0.0)	6 (5.3)	
Last test at VCT	68 (58.6)	2 (66.7)	66 (58.4)	0.776
At least one HIV test within last year	85 (73.3)	3 (100)	82 (72.6)	0.951
Positive HBV status at first cART	14 (12.1)	0 (0.0)	14 (12.4)	0.996
Positive HCV status at first cART	3 (2.6)	0 (0.0)	3 (2.7)	0.999
Positive syphilis status at first cART	26 (22.4)	1 (33.3)	25 (22.1)	0.951
Partner tested for HIV	42 (36.2)	2 (66.7)	40 (35.4)	0.92
Partner HIV positive	33 (28.4)	2 (66.7)	31 (27.4)	0.18
Always using condom with stable partners	56 (48.3)	1 (33.3)	55 (48.7)	0.605
Always using condom with casual partners	66 (56.9)	1 (33.3)	65 (57.5)	0.421
Sex on drugs or alcohol	24 (20.7)	1 (33.3)	23 (20.4)	0.59
Time to linked to care				
< 1 month	89 (76.7)	3 (100)	86 (76.1)	0.996
1–3 months	15 (12.9)	0 (0.0)	15 (13.3)	
>3 months	12 (10.3)	0 (0.0)	12 (10.6)	
HIV Subtype				
A/CRF/OTHER	13 (11.2)	0 (0.0)	13 (11.5)	0.998
B	87 (75.0)	3 (100)	84 (74.3)	
Unknown	16 (13.8)	0 (0.0)	16 (14.2)	
Year of first cART				
2010/11	24 (20.7)	1 (33.3)	23 (20.4)	0.988
2012	28 (24.1)	1 (33.3)	27 (23.9)	
2013	39 (33.6)	1 (33.3)	38 (33.6)	
2014/15	25 (21.6)	0 (0.0)	25 (22.1)	
cART regimen type				
PI	65 (56.0)	2 (66.7)	63 (55.8)	0.968
NNRTI	44 (37.9)	1 (33.3)	43 (38.1)	
InSTI	7 (6.0)	0 (0.0)	7 (6.2)	
Positive HLA B5701	5 (4.3)	0 (0.0)	5 (4.4)	0.981
Median(IQR)				
Age in years	31.6 (28.1, 37.3)	24.5 (23.3, 36.5)	31.6 (28.3, 37.4)	0.189
HIV RNA (log10 copies/ml)	4.5 (3.9, 5.1)	5.1 (4.7, 5.7)	4.5 (3.9, 5.1)	0.122
Lymphocytes CD4+ count (cells/mm3)	352.5 (259.5, 415.5)	523.0 (224.0, 574.0)	352.0 (265.0, 405.0)	0.361
Number of casual partners (1 year)	3.5 (0.0, 3.5)	3.5 (1.0, 3.5)		0.823
Number of partners (lifetime)	15.5 (8.0, 35.5)	15.5 (3.5, 35.5)	15.5 (8.0, 35.5)	0.746
Time to linked to care (years)	0.0 (0.0, 0.1)	0.0 (0.0, 0.1)	0.0 (0.0, 0.1)	0.146

Unadjusted hazard ratios (HR) between both preclinical and clinical factors and viral suppression are shown in Table 2. Those of bisexual orientation had an almost a threefold higher chance of achieving viral suppression relative to those of homosexual orientation (HR 2.97 95% CI 1.27, 6.96). Those with no reported history of sex on drugs and alcohol (HR 1.63 95% CI 1.02, 2.61), HLA B5701 genotype (HR 5.12 95% CI 1.98, 13.26), later year of starting cART and a non-PI based regimen at the time of starting cART also had higher chance of achieving viral suppression. Unknown syphilis (HR 0.46 95% CI 0.25, 0.85), and a higher HIV RNA level (HR For a tenfold increase: 0.61 95% CI 0.49, 0.75) were associated with lower chance of achieving viral suppression. Those who had one or more HIV tests within the last year (*P* = 0.078), of unknown HCV (*P* = 0.168) and HBV status (*P* = 0.064), relative to negative status relative to no disease were borderline associated with higher chance of viral suppression, and were also included in multivariate models.

**Table 2:** Unadjuste and adjusted hazard ratios for time to first viral suppression defined as HIV RNA < 50 after starting cART

Variable	Univariate			Multivariate		
	HR (95% CI)	*P* value	*P* value	HR (95% CI)	*P* value	*P* value
Age at starting cART						
Per year older	0.86 (0.67, 1.10)	0.218	0.218	0.77 (0.54, 1.09)	0.138	0.138
Sexual orientation						
Heterosexual	0.89 (0.49, 1.62)	0.698	0.037	0.98 (0.44, 2.17)	0.964	0.007
Homosexual	Reference			Reference		
Bisexual	2.97 (1.27, 6.96)	0.012		4.54 (1.76, 11.68)	0.002	
Number of tests within last year						
None	Reference		0.078	Reference		0.13
1 or more	0.69 (0.45, 1.04)	0.078		0.68 (0.41, 1.12)	0.13	
Year of first cART						
2010/11	Reference		0.011	Reference		0.316
2012	1.45 (0.82, 2.58)	0.203		1.80 (0.93, 3.50)	0.083	
2013	1.55 (0.90, 2.68)	0.116		1.50 (0.81, 2.77)	0.195	
2014/15	2.74 (1.50, 5.01)	0.001		1.22 (0.56, 2.66)	0.614	
HBV status at first cART						
Yes	0.84 (0.48, 1.49)	0.554	0.183	1.02 (0.47, 2.21)	0.957	0.708
No	Reference			Reference		
Unknown/missing	0.59 (0.33, 1.04)	0.07		0.76 (0.39, 1.49)	0.432	
HCV status at first cART						
Yes	0.77 (0.24, 2.45)	0.663	0.168	1.95 (0.48, 7.86)	0.347	0.642
No	Reference			Reference		
Unknown/missing	0.47 (0.22, 1.04)	0.064		1.12 (0.43, 2.92)	0.815	
Syphilis status at first cART						
Yes	0.83 (0.52, 1.31)	0.422	0.043	0.91 (0.55, 1.49)	0.702	0.003
No	Reference			Reference		
Unknown/missing	0.46 (0.25, 0.85)	0.013		0.27 (0.13, 0.57)	< 0.001	
HIV RNA at starting cART						
Per tenfold higher	0.61 (0.49, 0.75)	< 0.001	< 0.001	0.56 (0.42, 0.75)	< 0.001	< 0.001
Regimen type						
PI	Reference		< 0.001	Reference		0.003
NNRTI	2.00 (1.34, 2.98)	< 0.001		1.56 (0.95, 2.57)	0.079	
INSTI	2.87 (1.30, 6.35)	0.009		5.03 (1.90, 13.32)	0.001	
Sex on drugs or alcohol						
No	1.63 (1.02, 2.61)	0.041	0.041	1.42 (0.83, 2.43)	0.199	0.199
Yes	Reference					
HLA B5701						
No/Unknown	Reference		< 0.001			
Yes	5.12 (1.98, 13.26)	< 0.001		3.97 (1.33, 11.85)	0.013	0.013

Variables with *P* < 0.1 in univariate analysis (listed above) and age were adjusted for in multivariate analyses, and the resulting adjusted HR (aHR) are shown in Table 2. Bisexual orientation relative to homosexual orientation (aHR 4.54 95% CI 1.76, 11.68), a non-PI based regimen (particularly strong for integrase inhibitors aHR 5.03 95% CI 1.90, 13.32) and HLA B5701 positive (aHR 3.97 95% CI 1.33, 11.85) remained associated with higher chance of viral suppression. Unknown syphilis status (relative to no syphilis, aHR: 0.27 95% CI 0.13, 0.57) and higher HIV RNA (aHR a tenfold increase: 0.56 95% CI 0.42, 0.75) remained associated with lower chance of viral suppression. No association remained between number of HIV tests within the last year, year of starting cART, and reported sex on drugs or alcohol after adjustment for other factors (all *P* > 0.05).

Of the 116 people who started cART, 17 (15%) persons stopped their first regimen and 15 of 17 (13%) who stopped started a second regimen within 7 days. A summary of those who stopped their initial regimen is shown in Table 3. Of the 65 people who started on PI based regimen, 10 (15%) stopped treatment, followed by 7 (16%) of those who started NNRTI based regimen, and none of those who started InSTI. Reasons for stopping NNRTI included four (57%) due to hypersensitivity, two (29%) due to patient decision/non adherence, and one (14%) due to toxicity. Most (80%) of those who stopped PI were due to toxicity, one (10%) due to patient decision/non adherence and another one (10%) due to simplification of regimen. Of the two people who did not start a second regimen within 7 days of stopping, one was due to toxicity and one was due to patients decision or non-adherence (Table 3). Further information regarding specific toxicities was not available.

**Table 3:** Number of persons who stopped first regimen and reasons for stopping

	InSTI		NNRTI		PI		Total	
	*N*	%	*N*	%	*N*	%	*N*	%
Started treatment	7	100	44	100	65	100	116	100
Stopped or swapped regimen (ALL)	0	0	7	16	10	15	17	15
Reason for stopping *N* (% of those who stopped)								
ARV toxicity	0	0	1	14	8	80	9	53
ARV failure	0	0	0	0	0	0	0	0
Patient’s decision or non-adherence	0	0	2	29	1	10	3	18
Other or unknown	0	0	0	0	1	10	1	6
Hypersensitivity	0	0	4	57	0	0	4	24
Stopped regimen and did not restart	0	0	1	14	1	10	2	12
Reason for stopping *N* (% of those who stopped)								
ARV toxicity	0	0	1	100	0	0	1	50
Patient’s decision or non-adherence	0	0	0	0	1	100	1	50

## Discussion

In our study, four in ten people who tested HIV positive at the voluntary settings were not linked to care, but almost all those who were linked to care started cART and achieved rapid viral suppression during follow-up. In addition, most of the persons who started treatment in our study remained on the first cART regimen during the observation time.

Viral suppression remains the core factor in achieving treatment aims, namely reduction in morbidity/mortality and in HIV transmission. Therefore, we have investigated factors associated with the rate of viral suppression and identified bisexual orientation, HLA B5701 positivity and cART regimen to be significantly associated with faster rate of viral suppression. In earlier TAK analyses bisexual orientation was shown to be negatively associated with linkage to care [9]. In the survey study of sexual behaviours in 13 European cities, including Warsaw, men who have sex with men and women tended to be less open about their own sexual attraction as compared to men who have sex with men only. Furthermore, 60% of participants declared they would not openly admit their attraction to men [12]. This indicates that bisexual persons cannot be considered a homogeneous group, in terms of social behaviours. The association between bisexual orientation and faster viral suppression in the current study might be underpowered, as this group is represented by merely six persons in the studied population, and likely to disappear with a larger sample.

Recently there is a trend in focusing on key populations, defined as persons more likely to acquire or transmit HIV and it is considered that their engagement in prevention and educational programs is necessary for achieving better control over the HIV epidemic [13, 14]. The findings of the TAK project underlines that educational programs for the general population should not be omitted in order to decrease stigma and improve linkage to care and antiretroviral therapy intake. Although hetero- and bisexual mode of HIV transmission is far less frequent than homosexual, these two groups are at higher risk of not being linked to care thus remaining at risk of disease progression and HIV transmission [15]. In countries with high HIV fear and stigmatisation many persons with risky behaviour do not consider themselves at risk and do not spontaneously seek for information about HIV [16, 17].

In our study, both InSTI and NNRTI based regimens were associated with over twice faster rate of viral suppression as compared to PI based regiment, even after adjusting for other factors. The InSTI based regimen has been shown to have better efficacy and tolerability with lower treatment discontinuation rate as compared to PI-based regimens [18, 19]. The findings on NNRTI in our study almost in all cases relate to rilpivirine co-formulated with emtricitabine and tenofovir in one pill. Therefore, the observed faster suppression rate may reflect better adherence to one-pill regimen [20]. The impact of HLA B5701 is well in line with our earlier findings showing faster viral suppression after starting cART in persons with the presence of this allele, as well as with studies of long-term nonprogressors [21–23]. However, it needs to be underlined that other non-routinely tested HLA alleles, such as HLA B27 may play a role in natural control over viral replication.

Furthermore, we have identified a slower rate of viral suppression in persons with higher baseline HIV RNA, which is a well identified factor for worse or slower response to cART [24]. The unknown syphilis status, indicating ‘no-show’ to test, may also reflect worse compliance to physicians counselling, also resulting in suboptimal adherence and subsequent slower viral suppression in this group of patients [25].

Although genotyping is routinely performed in most of patients before starting cART these data were not collected in this study. Therefore, we cannot determine the role of drug resistance in response to treatment. However, Poland is a country with low prevalence of primary HIV drug resistance [26, 27]. In addition, our data are confirmed by other Polish analyses showing an initial response to first cART of over 90% [4].

There are some limitations of this study which need to be mentioned. Due to short time of follow-up, we were not able to investigate longer term adherence to cART and retention in care. This is an important issue in public health and with increasing follow-up in TAK cohort we plan to investigate it. Secondly, most of our study population is men having sex with men and our findings may not be generalisable to populations with higher representation of women or injecting drug users. In addition, it is important to note that linkage to care was comparatively low compared to the proportion starting cART or responding to cART, and it is likely that patients linked to care have self-selected to engage in HIV care, and the high proportions starting cART and responding to cART reflect this. Finally our multivariate model includes 18 estimated parameters and is based on a sample size of 113 individuals, therefore we cannot completely rule out the possibility that this model has been over-fitted. In addition, given that only three people failed to achieve virological suppression it needs to be underlined that the power to detect differences between the groups might be limited.

Viral suppression is required to prevent sexual HIV transmission, therefore the shortest time to achieving viral suppression provides the shortest window of opportunities for onward disease transmission [1]. In this perspective, better understanding of factors influencing time to viral suppression is crucial for controlling the epidemic [28]. In our earlier work, we have shown that the percentage of patient linked to care did not imrpove over calendar years, while the time to starting treatment shortened and the percentage of patients who started cART within 1 year from their first visit in the clinic increased [9]. Here we present that not only the time to starting cART, but also the time to achieving supressed viremia has improved over recent years. This can be contributed to substantial change in the understanding of early cART benefit resulting in physicians initiating treatment irrespective of CD4 count [29, 30]. In addition, wider access to InSTI and one tablet regimens in Poland has facilitated the availability of regimens with better tolerability and reduced pill burden, which may partially contribute to this effect. As a result, the relative benefit received from being linked to care, defined as decreasing morbidity and HIV transmission risk, has substantially increased over the recent years.

However, looking at the TAK cascade of care in a step-wise perspective we can see a necessity for improvement from the public health perspective. The current healthcare system in Poland is ready to respond to the HIV epidemic with early treatment and high suppression rates, but interventions increasing the number of people who are aware of their HIV status and assuring they are under specialist care and engage with such care should be a priority. To achieve any significant impact on the epidemic, scaling up HIV testing and linkage to care followed by timely cART initiation must be considered as a holistic action. The modelling study by Phillips et al. suggests that using condoms and effective cART had only a limited effect on the HIV epidemic, until combined with much higher rates of HIV testing [31]. This indicates the importance of close collaboration between often structurally separated agencies and stakeholders [6, 32]. The cascade of care is a helpful tool in this process allowing to both tailor and evaluate interventions. This warrants further investigation and supervision of linkage to care.

## References

[1] Cohen MS, Chen YQ, McCauley M, Gamble T, Hosseinipour MC, Kumarasamy N (2011). Prevention of HIV-1 infection with early antiretroviral therapy. N Engl J Med.

[2] Kowalska JD, Wojcik G, Rutkowski J, Ankiersztejn-Bartczak M, Siewaszewicz E (2017). Modelling the cost-effectiveness of HIV care shows a clear benefit when transmission risk is considered in the calculations—a message for Central and Eastern Europe. PLoS One.

[3] UN AIDS. 90-90-90 An ambitious treatment target to help end the AIDS epidemic. http://www.unaids.org/sites/default/files/media_asset/90-90-90_en_0.pdf Accessed 16 Nov 2016.

[4] Parczewski M, Siwak E, Leszczyszyn-Pynka M, Cielniak I, Burkacka E, Pulik P (2017). Meeting the WHO 90% target: antiretroviral treatment efficacy in Poland is associated with baseline clinical patient characteristics. J Int AIDS Soc.

[5] ECDC. Thematic report: HIV continuum of care. Available at: http://ecdc.europa.eu/en/publications/publications/dublin-declaration-continuum-of-care-2014.pdf. Accessed on 25 November 2016.

[6] Consolidated Strategic Information Guidelines for HIV in the Health Sector. WHO May 2015. http://www.who.int/hiv/pub/guidelines/strategic-information-guidelines/en Accessed 5 Nov 2015. 26110192

[7] Kowalska JD, Oprea C, de Witt S, Pozniak A, Gokengin D, Youle M (2016). Euroguidelines in Central and Eastern Europe (ECEE) conference and the Warsaw Declaration—a comprehensive meeting report. HIV Med.

[8] Ankiersztejn-Bartczak M, Firlag-Burkacka E, Czeszko-Paprocka H, Kubicka J, Cybula A, Horban A (2015). Factors responsible for incomplete linkage to care after HIV diagnosis: preliminary results from the Test and Keep in Care (TAK) project. HIV Med.

[9] Kowalska JD, Shepherd L, Ankiersztejn-Bartczak M, Cybula A, Czeszko-Paprocka H, Firlag-Burkacka E (2016). Poor linkage to care despite significant improvement in access to early cART in Central Poland—data from Test and Keep in Care (TAK) Project. PLoS One.

[10] Shepherd LA-BM., Cybula A, Czeszko-Paprocka H, Firląg-Burkacka E, Horban A, Mocroft A, Kowalska JD. Poor linkage to care despite significant improvement in access to early cART—data from Test and Keep in Care (TAK) project. In: 15th European AIDS conference. Barcelona 21–24 October 2015. Abstr. Nr. PS 8/4. 10.1371/journal.pone.0162739 PMC5053409 27711159

[11] PTN AIDS. Zasady opieki nad osobami zakażonymi HIV. 2016 http://www.ptnaids.pl/pliki/zalecenia_2016_uzupelnione.pdf. Accessed 23 Apr 2016

[12] Mirandola M, Gios L, Sherriff N, Pachankis J, Toskin I, Ferrer L (2017). Socio-demographic characteristics, sexual and test-seeking behaviours amongst men who have sex with both men and women: results from a bio-behavioural survey in 13 European cities. AIDS Behav.

[13] UNAIDS. UNAIDS strategy 2016–2021: on the fast-track to end AIDS. 2015. http://www.unaids.org/sites/default/files/media_asset/20151027_UNAIDS_PCB37_15_18_EN_rev1.pdf. Accessed 23 Apr 2016.

[14] Stahlman S, Hargreaves JR, Sprague L, Stangl AL, Baral SD (2017). Measuring sexual behavior stigma to inform effective HIV prevention and treatment programs for key populations. JMIR Public Health Surveill.

[15] Christopoulos KA, Das M, Colfax GN (2011). Linkage and retention in HIV care among men who have sex with men in the United States. Clin Infect Dis.

[16] The People Living with HIV Stigma Index (STIGMA Index) project. HIV-related stigma: late testing, late treatment. A cross analysis of findings from the people living with HIV Stigma Index in Estonia, Moldova, Poland, Turkey, and Ukraine. 2010. http://www.gnpplus.net/images/stories/Rights_and_stigma/2011_HIVStigma_Report_EN.pdf. Accessed 23 Nov 2013

[17] Rosinska M, Simmons R, Marzec-Boguslawska A, Janiec J, Porter K (2016). Relating HIV testing patterns in Poland to risky and protective behaviour. AIDS Care.

[18] Molina JM, Clotet B, van Lunzen J, Lazzarin A, Cavassini M, Henry K (2015). Once-daily dolutegravir versus darunavir plus ritonavir for treatment-naive adults with HIV-1 infection (FLAMINGO): 96 week results from a randomised, open-label, phase 3b study. Lancet HIV.

[19] Squires K, Kityo C, Hodder S, Johnson M, Voronin E, Hagins D (2016). Integrase inhibitor versus protease inhibitor based regimen for HIV-1 infected women (WAVES): a randomised, controlled, double-blind, phase 3 study. Lancet HIV.

[20] Gunthard HF, Saag MS, Benson CA, del Rio C, Eron JJ, Gallant JE (2016). Antiretroviral drugs for treatment and prevention of HIV infection in adults: 2016 Recommendations of the International Antiviral Society-USA Panel. JAMA.

[21] Proceedings of the 8th romanian national HIV/AIDS congress and the 3rd Central European HIV forum: Sibiu, Romania. 5–7 May 2016. BMC Infect Dis. 2016;16 Suppl 3:290. 10.1186/s12879-016-1480-8 PMC4928154 27356504

[22] Adland E, Hill M, Lavandier N, Csala A, Edwards A, Chen F (2017). Differential Immunodominance Hierarchy of Cd8 + T Cell Responses in Hla-B*27:05 and B*27:02-Mediated Control of Hiv-1 Infection. J Virol.

[23] Migueles SA, Sabbaghian MS, Shupert WL, Bettinotti MP, Marincola FM, Martino L (2000). HLA B*5701 is highly associated with restriction of virus replication in a subgroup of HIV-infected long term nonprogressors. Proc Natl Acad Sci USA.

[24] Flandre P, Pugliese P, Allavena C, Katlama C, Cotte L, Cheret A (2016). Comparative risk of failure of ABC/3TC or TDF/FTC based first-line regimens in patients with a high viral load. HIV Med.

[25] Remien RH, Dolezal C, Wagner GJ, Goggin K, Wilson IB, Gross R (2014). The association between poor antiretroviral adherence and unsafe sex: differences by gender and sexual orientation and implications for scale-up of treatment as prevention. AIDS Behav.

[26] Parczewski M, Leszczyszyn-Pynka M, Witak-Jedra M, Maciejewska K, Rymer W, Szymczak A (2015). Transmitted HIV drug resistance in antiretroviral-treatment-naive patients from Poland differs by transmission category and subtype. J Antimicrob Chemother.

[27] Stanczak GP, Stanczak JJ, Firlag-Burkacka E, Wiercinska-Drapalo A, Leszczyszyn-Pynkad M, Jablonowska E (2007). Transmission of HIV-1 drug resistance among newly diagnosed patients in Poland. Przegl Epidemiol.

[28] Phillips AN, Munderi P, Revill PA, El-Sadr WM, Lundgren JD (2014). Antiretroviral therapy recommendations for the global community: aspiration versus reality. AIDS.

[29] Geffen N, Aagaard P, Corbelli GM, Meulbroek M, Peavy D, Rappoport C (2015). Community perspective on the INSIGHT strategic timing of antiretroviral treatment (START) trial. HIV Med.

[30] Group ISToATS, Lundgren J, Babiker A, Gordin F, Emery S, Fatkenheuer G (2015). Why START? Reflections that led to the conduct of this large long-term strategic HIV trial. HIV Med.

[31] Phillips AN, Cambiano V, Nakagawa F, Brown AE, Lampe F, Rodger A (2013). Increased HIV incidence in men who have sex with men despite high levels of ART-induced viral suppression: analysis of an extensively documented epidemic. PLoS One.

[32] Kowalska JD, Grzeszczuk A, Pyziak-Kowalska K, Marzec-Bogusławska A, Rosińska M, Ankiersztejn-Bartczak M, Horban A (2017). Shaping the HIV epidemic in Poland—proceedings from the first Polish workshop on cascade of care in HIV. HIV AIDS Rev.

